# Implementing a behaviour change communication interaction for enhancing male involvement in maternity care among the Saharia Tribes in Gwalior District, Madhya Pradesh: a feasibility study

**DOI:** 10.1186/s12913-025-13099-5

**Published:** 2025-08-18

**Authors:** Tulsi Adhikari, Saritha Nair, Ashpinder kaur Grover

**Affiliations:** 1 Indian Council of Medical Research, Ansari Nagar, New Delhi, India; 2 Faculty of Medical Research, Academy of Scientific and Innovative Research (AcSIR), New Delhi, India; 3 Division of Policy & Communications, Indian Council of Medical Research, New Delhi, India

**Keywords:** Feasibility study, BCC, Maternity care services, ANC, PNC, Reproductive health, Male involvement, Gender role in health care, Cultural influences on health care

## Abstract

**Background:**

The Indian tribal population is varied, with a wide range of customs, ways of life, and cultural practices. However, there is one thing that all Indian tribal communities have in common: they have worse health indicators, a higher rate of illness and mortality, and very restricted access to medical care. Their health issues require extra consideration in the right setting (Salil, Health and Population Perspectives and Issues 23:61-70, 2000). Growth in the utilization of reproductive and maternal health services will not only curtail down the reproductive morbidities, but it will also reduce the child mortality (Sharma et. al, Utilization of health services and RCH status in Madhya Pradesh: a District Level Analysis. In Proceedings of National Symposium on Tribal Health 2011). Men’s participation in prenatal care, delivery and postpartum period is rarely found, especially among tribal communities, due to their economic instabilities and priorities. Also our health system does not promote the involvement of men in the maternal and child health care. Hence, there was a need felt for development of gender and community sensitive interventions package that could address the individual and the community health care facility level barriers of male involvement in utilisation of the maternal care services. Our study was an effort to determine the feasibility of implementing a behaviour Change Communication Interaction developed for improvement in utilisation of maternity care services through male participation among the Saharia Tribes in Gwalior District, Madhya Pradesh.

**Methods:**

The Study utilised a qualitative approach. Various activities were organised as a part of BCC, viz., Community mobilization, Campaign/Rallies, Interpersonal Communication-Drama & Mock Sessions, Face to Face counselling and Quiz etc. Action technique called Transect was used in order to know more about the environment and living of the people of Saharia Tribes in Gwalior District, Madhya Pradesh. Feasibility of the model was assessed by focusing on three main principles i.e., acceptability, integration and limited-efficacy testing.

**Result:**

Acceptability testing study reveal that BCC intervention was successfully accepted by intended individual- both targeted individuals and those involved in implementing programs. Integration approach reveal that no major change in infrastructure of Govt. Programmes and facilities is required. Instead, effective application relies on the engagement of key community members and local health service workers. Limited-efficacy testing reveals that there is a behavioural change in men’s perception of accompanying their spouse to the health centre; same was observed on the vaccination day in the village.

**Conclusions:**

The BCC intervention proved to be feasible to implement. The Proposed BCC interaction is feasible and accepted by both Programme stake holders and beneficiaries.

**Supplementary Information:**

The online version contains supplementary material available at 10.1186/s12913-025-13099-5.

## Background

The Indian tribal population is varied, with a extensive range of customs, ways of life, and cultural practices. However, there is one thing that all Indian tribal communities have in common: they have worse health indicators, a higher rate of illness and mortality, and very restricted access to medical care. Their health issues entail extra consideration in the right setting. The participation of men in maternal health services is critical for the overall growth of both mothers and their children. Many research findings show that when men are actively involved in maternal health, it strengthens the relationship within the family and also lowers the costs associated with health problems. The men who enthusiastically participate in reproductive activities are well informed about the govt. facilities, vaccination, post and prenatal care during pregnancy which improves the utilisation of these services by the pregnant women and further curtail down the rate of maternal mortality. Studies have also indicated that couples who have good communication about pregnancy are more likely to raise their children in a healthier environment [1, 2]. 

It has been revealed in various studies that men participation in maternal health is rarely found, reasons associated with it are lack of knowledge of RCH services, illiteracy, addiction of alcohol, financial crisis, family matters, loss of wage, health centre resource constraints, Behaviour of health centre workers, lack of interest of policy makers, male dominance, attitude of elders in the home, laziness, personal attitude towards antenatal services, lack of trust on health workers, domestic violence and various others. Considering these obstacles, numerous studies highlight the necessity for creating more effective interventions and feasibility studies to overcome these barriers and to enhance men’s participation in maternal health services [3]. 

One significant indicator to assess the status of healthcare in any nation is the maternal mortality ratio, or MMR. In the past 20 years, India has significantly decreased the number of maternal fatalities. The Maternal Mortality Ratio (MMR) in India was notably high in 1990, with 600 deaths per 100,000 live births, or nearly 1.5 lakh deaths annually. According to the estimates from Sample registration system (SRS), under-5 mortalities in India is 36/1000 live births(SRS,2018) and Neonatal Mortality Rate is 23/1000 live births(SRS,2018). MMR in the country has declined from 130 in 2014-16 to 97 in 2018-20 (SRS,2018-20) Over the former 27 years, there has been a 45% decrease on a global scale. To facilitate quality health care to pregnant women, various Programmes has been launched by Govt. of India including Janani Suraksha Yojana (JSY), Janani Shishu Suraksha Karyakaram(JSSK), Pradhan Mantri Surakshit Matritva Abhiyan(PMSMA), Surakshit Matritva Aashwasan (SUMAN), among others [4].

RCH services aims at combating and reducing the mortality rates of infant, mother and children. Numerous studies on Utilization of Health Services and RCH Services has been done which clearly depicts that districts that facilitate more RCH services also have better reproductive and child health. Therefore, growth in the utilization of reproductive and maternal health services will not only curtail down the reproductive morbidities, but it will also reduce the child mortality [5]. Implementation of interventions in the concept of good health and disease prevention found to be both efficacious and effective. Due to resource constraints all interventions cannot be tested for both efficacy and effectiveness and hence, feasibility studies play a profound role in determining whether an intervention should be recommended for efficacy testing. To make favourable health behaviour changes, Behaviour change communication (BCC) is used to provide revive messages and a supportive environment that encourages individuals and communities to enhance their knowledge about health [6].

BCC intervention plays a vital role in influencing the people’s social, environmental and organizational conditions as well as their preferences, notions and customs. However, there are many empirical evidences which reveal that BCC intervention is of massive importance in bringing changes in the behaviour of community. To curb down the child morbidity and mortality in poor communities Bangladesh Rural Advancement Committee (BRAC) initiated Improving Maternal neonatal and child survival (IMNCS) programme. The IMNCS programme introduced BCC interventions from its initiation in 2006. The motive of this study was to explore community perceptions of BCC interventions of the BRAC IMNCS programme in rural Bangladesh. BRAC is one of the largest NGOs in the world. The study revealed that the IMNCS BCC interventions had influenced both men and women to take health promoting decisions and seek maternal, neonatal and child health services. Observable and important changes such as improvement in healthy cord care practice, delayed bathing of the new-born and reduction of infant mortality was also observed between intervention and comparison districts [7–9]. 

The study of the Khairwar tribe of Madhya Pradesh shows that most of the males have lack of knowledge about the type of services provided and the number of ANC visits in different trimesters of pregnancy [10]. Further, they have an indifferent attitude towards it. Most of the deliveries are made at home. Our study on the development of the BCC Model for improving male participation in maternity care [11] suggested a lack of male participation, and despite the availability of health facilities near the villages, low uptake of RCH indicators was there among the Saharia tribal community in the Gwalior district of Madhya Pradesh. This specifies a significant gap in alertness and engagement with maternal health services. Addressing these challenges requires targeted interventions that enhance male participation and train the community about the significance of accessing healthcare facilities during pregnancy and childbirth.

A study on community perspective on men’s role in the utilization of maternal health care services among Saharia Tribes reveals that men seldom accompanied women for antenatal check-ups due to lack of knowledge, awareness, norms, beliefs, gender inequitable attitudes etc. There is a need for development of gender sensitive interventions that address the individual, community health care facility level barriers of male involvement [12]. Saharias, one of the primitive tribes, are mainly located in the Chambal division, i.e., Gwalior, Morena, Guna and Shivpuri. Their total population is over 2 lacs and is one of the poorest primitive tribe of Madhya Pradesh [13]. The main residential area of Saharia tribe is the forest of Shahabad which is spread from Rajasthan to Guna of Madhya Pradesh. Main source of income of Saharia Tribes is from agricultural work. They are generally influenced by Hindu culture; they worship many Hindu God and Goddesses and also celebrate various Hindu festivals. Our study was an endeavour to determine the feasibility of implementing a Behaviour Change Communication interaction developed for improvement in utilisation of Maternity care services through male participation among the Saharia Tribes in Gwalior District, Madhya Pradesh.

### Aims and objectives

The aim of the study was to determine the feasibility of implementing a behaviour Change Communication interaction for improvement in utilisation of Maternity care services through male participation among the Saharia Tribes in Gwalior District, Madhya Pradesh. The Maternity care services aims at providing at least three antenatal check-ups, immunization against tetanus, and iron and folic acid for anaemia management.

### Process prior to implementation of the BCC model

To know more about the environment and living, a participatory learning & action technique called Transect was used. Transect is an observatory walk through the residential area of a village, observing and making notes of the layout of the village, housing, drainage, backyards, infrastructure, (schools, shops, wells, electricity), etc. It helps to locate/assess, map, and analyze various aspects of the residential area of the village that normally go unnoticed. A village transect is different from the other transects as it has greater focus on the dwelling zone of the village. It also provides extensive coverage of the entire household and economic activities which would probably be otherwise go unnoticed.

BCC experts along with the two project staff conducted the face to face counselling session for getting the perceptions of the couples around male involvement in maternity care services, Couples seemed to be enthusiastic about the whole concept. Verbatim of the couple during house to house survey is as under

Such kind of activities were never performed before in the area. We are very optimistic about this activity.

One of the concerned raised by a woman in the village was substance abuse. The verbatim is as under.

“Most of the men in our village start having alcohol since from 9A.M. in the morning. How will they accompany us for health check-up”?

To explore more of local male tribes of the village they were called to assemble in a place where two-way communication could be held. A meeting was organized with all households at the Anganwadi to explain them the objective of the intervention in the area. Since it was open forum, opportunity was given to all male members and their female partners too to open up and share the views about existing health services available to them.

One of the men replied that “Accompanying women to the health center is not men’s job” its women’s job only”.

But there are few people in the village who were ready to work for the cause and one elderly men said “We are ready to motivate young couples for male involvement and for better maternity care”. Based on the information generated through the study and the quantitative and qualitative analysis, BCC intervention model was developed in consultation with the BCC expert [11].

### Methodology

There are number of assessment indicators for feasibility study but our study includes Acceptability, Integration and Limited-efficacy testing as described below [14]. 

#### Acceptability

 This widely adopted approach examines how the intended individual recipients—both targeted individuals and those involved in implementing programs—retort to the intervention.

#### Integration

This approach evaluates the extent of system change required to integrate a new program or process into an existing infrastructure or program. The documentation of change that occurs within the organizational setting or the social/physical environment as a direct result of integrating the new program can help to determine if the new project is truly feasible.

#### Limited-efficacy testing

Feasibility studies often assess interventions on a limited scale. Such assessments may be conducted in a convenience sample, with intermediate rather than final outcomes, with shorter follow-up periods, or with limited statistical power.

### Study design

The Study utilised a qualitative approach. BCC activities were planned initially to begin in one of the village for testing the feasibility of the model. Twenty male members from the village were selected for awareness training initially to engage them in the activities planned by the team and technical expert of BCC. The intervention was delivered for a period of one month. Pre-post awareness questionnaire was filled by the registered men. To check whether the BCC model might be integrated into the existing project few satisfaction interviews were also conducted among the programme delivery personnel.

### Participants

For the awareness training on RCH for Males, all men of 18 years or above age, who voluntarily agreed to participate, were enrolled for the 2-day training awareness programme during 16/12/2017 to 17/12/2017.

### Procedure

Various activities were organised as a part of BCC like Community mobilization, Campaign/Rallies, Interpersonal Communication-Drama & Mock Sessions, Face to Face counselling and Quiz etc. Action technique called Transect was used in order to know more about the environment and living of the people of Saharia Tribes in Gwalior District, Madhya Pradesh.

### Training of trainers

The social worker’s employees under the study were given 4 days vigorous training on development of BCC and doing feasibility study under the able guidance of BCC expert from National Institute for Public Cooperation and Child Development, New Delhi.

### Implementation of the BCC interaction for limited-efficacy testing

BCC strategy was worked out in one of the tribal District of Gwalior. As per the baseline data, the BCC activities were planned initially to begin in one of the village. Activities like Awareness Training on RCH for Males, Community Mobilization, Campaign/Rallies, Interpersonal Communication, Face to Face counselling and quiz etc. were organized as a part of BCC. BCC must play a role in awakening the community response to ensure community-based health services critical to maternal and new born health are available.

The awareness training programme for the male members of Saharia tribes was conducted at village in Dabra Block with the following agenda depicted in Table 1.

**Table 1:** Awareness training programme

Day 1	
Introduction about the programme	BCC expert
Pre-test (Regarding knowledge of RCH facilities (ANC/PNC/Delivery and Immunization)	Social worker under the project, ASHA, Anganwadi Worker
Discussion About Maternal and Child Health Facilities, ANC/PNC Delivery and Immunization	Child Development Public Officer (ICDS)
Role & Responsibilities of Husband towards RCH services Utilization	BCC expert +
Quiz and Prize Distribution	
Day 2	
Visit of Nearest health Centre	Could not be conducted due to strike
Discussion on Knowledge of Maternal and Child Health facilities provided by government. Barriers and Solutions for Male Participation	BCC expert, Social worker under the project and CDPO
Post test (Regarding knowledge of RCH facilities (ANC/PNC/Delivery and Immunization)	Social workers and CDPO

### Community mobilization

Community gathering with registered eligible couples as well as other senior village members, local community health care providers and field employee’s under the project was held in the village. BCC activities to enhance men’s participation in maternal and child health were discussed. The male and females presented themselves in the course of the meeting. They provided their perspective on the BCC intervention activities conducted by field staff, local health workers and the BCC expert from NIPCCD. Drama performance was also conducted by the male members and the local community health care providers with the aim to enhance male participation in RCH services, viz., ANC, PNC and immunisation. House-to-house visits were also undertaken to gather individual perspectives and concerns about improving men’s participation in the RCH.

### Campaigns/rallies

An awareness generation on RCH and the involvement of men was planned and organized in the region. Many people were involved in the campaign, such as Village leaders, AWW helpers, School Children, ANM, ICDS Supervisors, training team of BCC, Youth club members and SHGs along with school going adolescent girls. Campaign target areas were Maternal Health & Nutrition, Family planning, Child Health & Nutrition, Routine immunization, Hygiene & Sanitation, adolescent health & Nutrition and reduction of Alcohol consumption. A rally was arranged with all of the members present, and the volunteers went around the town delivering the messages that had been prepared in advance. Video coverage of campaign was done. The English translation of the messages is as under–

- 1. "The wife has always fulfilled her responsibilities; now it’s the men’s turn".
- 2. "Men who take care of their families are truly great".
- 3. "If mother and child are vaccinated, the family will have a happy environment".
- 4. "A responsible man recognizes his duty—take care of his wife and child".
- 5. "Our wife, our child—then why this distance? Wake up and think, men, isn’t health important?"

### Wage loss compensation and rewards for the active participants in the BCC programme

The male members registered in the village received compensation for lost wages for all the days spent in the BCC team. The male members of the village who supported the cause were given additional rewards. A total of rewards are divided into two categories, one is for active husbands, and the other is for active participation in the Drama. Five male members were selected from each category and rewarded in the form of backpacks and blankets. Awarded male members and two females (an elderly and an eligible female were interviewed and their views on BCC activities and male participation in MCH service facilities were taken.

## Results

**Fig. 1:**
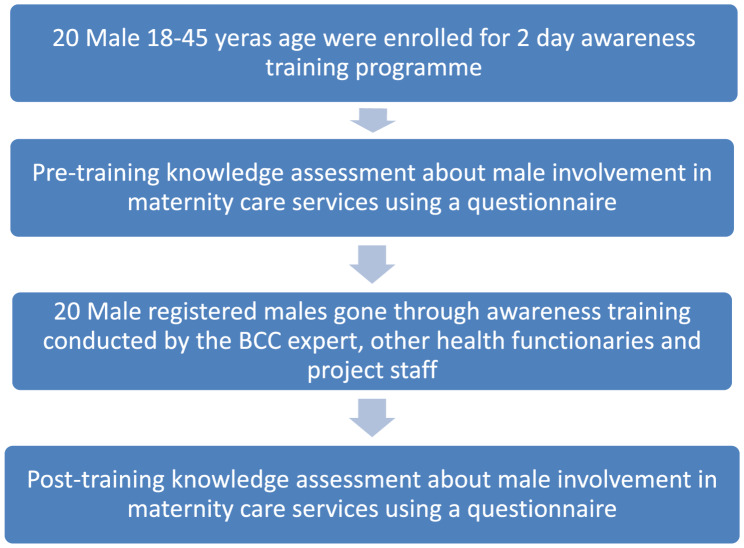
Consort Diagram of the Flow of participants of the 2-day training awareness programme

The awareness training programme for the male members of Saharia tribes was conducted at village in Dabra Block. The flow of participants of the 2-day training awareness programme is given in Fig. 1 below.

### Acceptability

#### Targeted individual’s reaction to the intervention

Community gathering with registered eligible couples as well as other senior village members, local community health care providers and field employee’s under the project was held in the village. The male and females presented themselves in the course of the meeting. Men seem to be more vocal in expressing their willingness to take part in the maternal and child health services being provided by the govt. The village’s elders also supported male involvement. Couples appeared to be passionate about the entire idea. They had been of the view that such forms of activities were by no means being performed before, with-in the area and were very much optimistic about impact of BCC activity. But they had been worried concerning the bad behaviour the various male viz. alcoholism, which of their opinion is going to be one of the boundaries in enhancing the male involvement in MCH services.

Couples enthusiastically expressed their views on the BCC intervention activities being carried out by the field staff, local health workers and expert from NIPCCD. They were of the view that these kinds of activities will enhance the role of men in maternal health care and will bring fruitful consequences in favour of women.

#### Reaction of individuals’ involved in implementing programs

The individuals (health providers) involved in the implementation of the BCC model programme, also some of the key informants, were asked about their views on the activities under the BCC programme using a feedback form. The respondents were asked about the benefits and process of implementation of the activities under the proposed BCC model programme.

The respondent believed that the proposed knowledge and awareness program would lead to greater awareness of government programs and health facilities provided under the programs. It would change the behaviour of society and would also improve male participation and the health of both the mother and the child in general. It would also reduce maternal and infant morbidity and mortality in society. The awareness program would also improve mutual trust and coordination between couples in the community.

#### Feedback on community level activities under the BCC model

The respondent believed that the activities proposed at the community level would improve the attitudes of individuals and the community at large towards the husband’s involvement in maternal and child health activities. The proposed activities would create an environment conducive to the participation of men in maternal and child health and the male members of the village would no longer be ashamed to accompany their wives to the hospital for ANC/PNC, vaccination and delivery. This would improve the proportion of institutional deliveries and have a better impact on maternal and child health. The Nukkad Games of the BCC program would support the process of improving male participation in maternal and child health.

#### Feedback on quiz conducted with registered men

A quiz was conducted before and after the intervention of the BCC Model with the help of stakeholder groups and ANW. Various questions like where the delivery of pregnant women should be done? Did Husband should accompany his wife to health centre? Is it Mandatory to vaccinate new born and expected mothers? Do pregnant women need a nutrition regime as opposed to a normal diet? Did u felt ashamed of accompanying pregnant women to health centre? If you did not accompany your wife to health centre, did she able to get more beneficial health services? Will you give time to your wife and new born after delivery? The questionnaire was developed and used for assessing the pre and post knowledge about male involvement in maternity care services. The questionnaire used is attached as supplementary file.

#### Outcome of the quiz

**Fig. 2:**
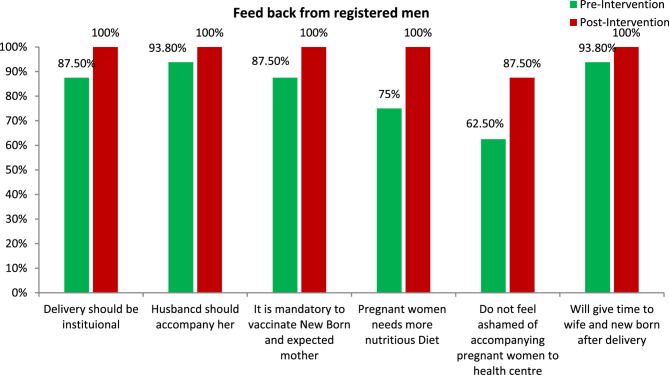
Short term impact of the BCC intervention on the knowledge and perception of registered men

Drastic change was seen after the intervention. 100% of the men agreed that delivery should take place in a health centre, that the husband should accompany his wife to the health centre, that both the new born and the expectant mother must be vaccinated, and that they understood the importance of a proper nutritious diet and accompanying their wife to the health centre. Before the intervention, 87.5% of men stated that delivery should be done institutional. Only 62.5% of men felt no shame in accompanying their wives to the health center. After the intervention, this increased to 87.5%.“Outcome of the pre and post intervention is depicted in Fig. 2.

#### Feedback on home visits for couple counselling

The respondent believed that the proposed home visits for couple counselling would improve the knowledge of the families in the society about the benefits of the male participation in maternal and child health. Through this process the problems and believes of couples could be handled individually; this would help in accelerating the process of male participation.

### Integration

This approach assesses the degree of system change required to integrate the BCC model program with the existing infrastructure. And according to the components of our BCC model, the required system change is minimal. The various components of the Model BCC require different system changes.

1. Based on the awareness rallies and Nukkad Nataks (Street Plays) that are part of the community participation in the behavior change communication model, no major infrastructure change is needed, only the participation of key people in society and worker’s local health services is required.
2. The awareness program for the of eligible men and women group meetings, and the home visits of local health workers requires an additional component, namely, the participation of men in maternal and child health in the duties of the local health workers and improved level of commitment from them for success of the BCC model.

### Limited effectiveness testing

The short-term effect of the BCC intervention carried out in one of the Village was visible in registered couples as part of the awareness program. The projected intervention is implemented in the area for one month (14th Dec 2017 to 13th Jan 2018), and the BCC intervention short-term impact was evaluated after one month. To assess impact, a community meeting of project researchers was held in the village with registered eligible couples along with other older village members, local health care providers and field workers as part of the project. Some of the villagers were also interviewed during the short-term impact assessment.

1. Villagers believed that male members of society should be actively involved in the use of maternal and child health services, namely prenatal care, maternal delivery and vaccination. Male members must accompany their wives to prenatal care visits, vaccination visits, and hospital deliveries. They must ensure that the pregnant woman takes 100 IFA tablets provided by the health center.
2. The practice in the past has been to give birth at home and for institutional deliveries and for ANC/PNC visits, generally the mother-in-law or some elderly women accompanied the woman to the health center. But after the awareness program and other BCC activities in the village, their mind changed and most of the people were in favor of her husband accompanying the woman to the health center. They believed that the amount of logistical support that a husband can provide to the wife cannot be provided by anyone else, including the elderly woman in the home.
3. During this brief period, there was a visible change in men’s perception of accompanying their spouse to the health center. A great change was observed in the presence of men on the day of vaccination.

## Discussion

The aim of the study was to determine the feasibility of implementing a behavior change communication interaction for improvement in utilization of maternity care services through male participation among the Saharia tribes in Gwalior District, Madhya Pradesh. BCC activities were planned initially to begin in one of the villages for testing the feasibility of the model. The study explored three major areas of the feasibility study, i.e., limited efficacy testing, acceptability, and integration.

From the acceptability testing, the study reveals that by implementing the BCC interaction, people became more aware of facilities provided by the government. Couples seem to be more enthusiastic about the entire idea. They were of the view that such kinds of activities were never conducted in their area. But they had been worried concerning the bad behavior of the various males, viz., alcoholism, which, in their opinion, is going to be one of the boundaries in enhancing the male involvement in maternity care services. The respondents were of the view that due to intervention mortality rate among women will decline, and men will no longer feel ashamed to accompany their wives to the health center.

The integration approach reveals that no major infrastructure change is needed; only the participation of key people in society and workers in local health services is required. For this impact to take place, the support of state-level health delivery system-level stakeholders is a must, and rigorous effort needs to be made. The short-term impact of the BCC intervention shows that there is a change in men’s perception of accompanying their spouse to the health center. A great change was observed with the presence of men along with their wives and children on the day of vaccination. These results are in line with the results of earlier studies, which reveal that behavioral interventions have fruitful results. Various studies on tribal populations reveal the same fact: alcoholism is identified to be one of the biggest impediments to men’s involvement in maternal health care [12]. A study on mortality among Saharia reveals that poor eating habits, negligence of iron, calcium, vitamins, and maternal care during pregnancy from the inception of the pregnancy to its termination are all factors that contribute to maternal mortality among Saharia women [15]. Our study, which used the BCC model, resulted in a variety of changes in the behavior of elderly people, both men and women, as well as a shift in their attitudes on men’s engagement in maternal care. People become more knowledgeable about the government’s numerous services. This increased awareness has led to greater participation in maternal health programs, ultimately contributing to improved health outcomes for both mothers and infants. Furthermore, the involvement of men in maternal care has fostered a supportive environment that emphasizes shared responsibility in health and childcare.

Moreover, the health status of tribal women in India, their access to employment, and men’s role in the utilization of maternal health care services among Saharia tribes reveal that there is a need for the development of gender-sensitive interventions that address the individual, community health care facility-level barriers of male involvement [12, 16]. Our study, which used the BCC model, focused on increasing male engagement in the utilization of maternity care services. Men who were previously hesitant to accompany their wives to the health center now understand the value of ANC and PNC visits after participating in the intervention program. This increased understanding has not only improved their wives’ access to essential maternity care services but has also fostered a supportive environment for family health decisions. As a result, we observed a significant enhancement in overall maternal and child health outcomes within the community.

### Limitations

In the proposed BCC interaction, there was a provision to visit the nearest health center, but due to a strike on the scheduled day of the visit, we were unable to fulfill the task; hence, this element was eliminated from the study. In the proposed BCC interaction, it was planned that co-workers would conduct a meeting with all the stakeholders to discuss the BCC activities, but due to the non-availability of the stakeholders, this component was modified, and meetings were held with stakeholders based on their availability rather than necessarily with all of them at once.

As the BCC intervention is a customized communication package for a specific group, and in our study it is customized and developed for the tribal population of Saharia tribes in Madhya Pradesh. The proposed BCC interaction feasibility study was customized for two blocks of the Gwalior district, Madhya Pradesh. Hence it can be generalized for the whole tribal community of Madhya Pradesh state only. This targeted approach ensures that the unique cultural and social dynamics of the Saharia tribes are taken into account, fostering more effective communication and engagement. Consequently, the insights gained from this study can serve as a valuable foundation for similar initiatives aimed at improving health outcomes within other tribal communities across Madhya Pradesh.

### Future endeavours

After testing the feasibility of the Proposed BCC intervention, we have initiated a community trial for assessing the impact of the BCC on male involvement and utilisation of maternal care services in four districts of Madhya Pradesh.

## Conclusion

Motive of our study was to determine the feasibility of implementing a behaviour Change Communication interaction for increasing male engagement in utilisation of maternity care services among the Saharia Tribes in Gwalior District, Madhya Pradesh.

The BCC intervention proved to be quite fruitful, as changes in the knowledge and attitude of both men and women as well as the elderly in the hamlet, were noted. Men no longer hesitate to.

acccompany their wives to ANC, Institutional delivery and PNC visits after intervention. They become more aware of the facilities provided by the Govt. No major changes to the infrastructure are required, just the participation of key people in society and services of local health workers is required. Those who participated in the implementation of the program believed that it would also reduce maternal and infant morbidity and mortality in society. In a nutshell, this type of study can significantly change people’s lives/attitudes. The Proposed BCC interaction is feasible and accepted by both Programme stake holders and beneficiaries.

## Supplementary Information

Supplementary Material 1.

## Data Availability

Data used or analyzed during the study are available on reasonable request from the corresponding author.

## Abbreviations

BCC: Behaviour change communication
RCH: Reproductive and child health
BRAC: Bangladesh Rural Advancement Committee
ANC: Antenatal care
PNC: Postnatal care
IMNCS: Initiated Improving Maternal Neonatal and Child Survival
