# Supplementary material for: Implementing a behaviour change communication interaction for enhancing male involvement in maternity care among the Saharia Tribes in Gwalior District, Madhya Pradesh: a feasibility study
Source: BMC Health Serv Res. 2025 Aug 18;25:1100. doi: 10.1186/s12913-025-13099-5 (PMC12362996; doi:10.1186/s12913-025-13099-5)
Supplement: Supplementary file 1 — Supplementary Material 1. [file 12913_2025_13099_MOESM1_ESM.docx]

**Implementing a Behaviour Change Communication interaction for enhancing male involvement in maternity care among the Saharia Tribes in Gwalior District, Madhya Pradesh: a feasibility study**

**Assessment of the pre and post knowledge about male involvement in maternity care services**

Q1. Where the delivery of pregnant women should be done? (Institutional-1, Home-2)

Q2. Did Husband should accompany his wife to health centre? ( Yes-1, No-2)

Q3. Is it Mandatory to vaccinate new born and expected mothers? ( Yes-1, No-2)

Q4. Do pregnant women need a nutrition regime as opposed to a normal diet? ( Yes-1, No-2)

Q5. Did u felt ashamed of accompanying pregnant women to health centre? ( Yes-1, No-2)

Q6. If you did not accompany your wife to health centre, did she able to get more beneficial health

services? ( Yes-1, No-2)

Q7. If you did not accompany to health centre will you be able to fulfil your responsibility as a

husband ( Yes-1, No-2)

Q8. Will you give time to your wife and new born after delivery? ( Yes-1, No-2)
